# High Corticosterone Affects Somite Development During Early Avian Embryogenesis

**DOI:** 10.3390/biom16071014

**Published:** 2026-07-11

**Authors:** Deeksha Aarti Nursimulu, Anita Heiß, Jiazhao Song, Hanne Jahns, Prity Pugo-Gunsam, Regine Schneider-Stock

**Affiliations:** 1 Department of Biosciences & Ocean Studies, Faculty of Science, University of Mauritius, Réduit, Moka 80837, Mauritius; deeksha.nursimulu1@umail.uom.ac.mu (D.A.N.); gunsamp@uom.ac.mu (P.P.-G.); 2 Experimental Tumorpathology, Institute of Pathology, Universitätsklinikum Erlangen, Friedrich-Alexander Universität Erlangen-Nürnberg, Universitätsstr. 22, 91054 Erlangen, Germanyjiazhao.song@extern.uk-erlangen.de (J.S.); 3 Pathobiology Section, School of Veterinary Medicine, University College Dublin, Belfield, D04 W6F6 Dublin, Ireland; hanne.jahns@ucd.ie; 4 Comprehensive Cancer Center Erlangen-EMN (CCC ER-EMN), Bavarian Cancer Research Center (BZKF), 91054 Erlangen, Germany

**Keywords:** stress hormone, somites, embryonic development, chick embryo model

## Abstract

Maternal stress has been associated with altered embryonic and fetal development, yet the mechanisms by which stress hormones influence early developmental processes remain poorly understood. Early embryogenesis is particularly sensitive because it establishes the vertebrate body plan through tightly regulated events such as somitogenesis. Disruptions during this developmental window may therefore compromise normal morphogenesis and tissue organization. In the present study, we investigated whether elevated acute corticosterone exposure affects somite phenotype and Sonic hedgehog (SHH) signalling during early embryogenesis using the avian chick embryo model. Fertilized specific pathogen-free (SPF) chick eggs were incubated until Hamburger–Hamilton stage 12 (approximately 48 h of incubation) and subsequently injected with 15 µg corticosterone into the yolk sac to mimic acute high-stress conditions for 4 h. Macroscopic examination revealed increased inter-somitic distances between the first five visible pairs of somites in corticosterone-treated embryos compared with controls, suggesting altered somite organization. In addition, histological assessment indicated that corticosterone-treated embryos displayed occasional cells with morphological features consistent with cellular degeneration or cell death. Molecular analysis demonstrated significant downregulation of *SHH* and *GLI1* expression, indicating potential impairment of the SHH signalling axis during somitogenesis. The expression of *TGFβ4* was also reduced, whereas *HIF2α* expression was elevated. These findings suggest a possible association between stress-induced hypoxic responses and altered developmental signalling, warranting further investigation. To the best of our knowledge, this is among the first in vivo studies to examine the relationship between elevated corticosterone exposure and SHH-associated somite development during early embryogenesis.

## 1. Introduction

The prenatal period is recognized as a critical window of development, because any physiological, emotional, or psychological alterations occurring during this time may significantly affect the overall development, growth and health of the fetus [1]. Among the various factors influencing this stage of gestation, maternal stress has emerged as a particularly important determinant of fetal outcome [2]. Heightened stress levels can disrupt the hormonal balance necessary for a healthy pregnancy, primarily by interfering with the homeostatic release of cortisol, the principal hormone associated with stress [1]. Dysregulation of the maternal hypothalamic–pituitary–adrenal (HPA) axis has been shown to increase the transfer of lipophilic stress hormone from the maternal to the foetal compartment, which may be associated with alterations in foetal development [3,4]. Additionally, apart from hormones, the composition and signalling properties of amniotic fluid (AF) and nascent cerebrospinal fluid (CSF) in the developing mouse brain are dynamic and stage specific [5]. Taken together, these insights underline how changes in maternal signalling and hormonal transfer may alter the foetal milieu and subsequent development.

During early embryogenesis, the timing and regulation of segmentation and patterning are significant in establishing the vertebrate body plan, as periodically arranged somites provide the blueprint for the segmentally organized musculoskeletal, vascular and peripheral nervous systems [6]. Translating this structural blueprint into specialized tissues requires the coordinated action of various signalling molecules. Working in concert with other signalling pathways such as the Wnt and Notch signalling pathways [7], the morphogen Sonic hedgehog (SHH), which is secreted by the notochord and the neural tube floor plate, is essential for growth, patterning and morphogenesis [8]. Additionally, several studies have also demonstrated interactions between hypoxia-inducible factors and Hedgehog signalling pathways during development and tissue homeostasis [9,10,11,12].

Dysregulation of SHH signalling has been linked to various developmental disorders, including the absence or malformation of ventral neuronal subtypes [13,14]. Given the sensitivity of the developing fetus to molecular perturbations, understanding how elevated stress hormone levels influence SHH activity is crucial. Glucocorticoid receptors are expressed during early embryonic development and mediate transcriptional responses to elevated glucocorticoid levels [15]. Consequently, excessive corticosterone exposure during critical developmental windows may interfere with signalling pathways involved in tissue patterning and morphogenesis. Therefore, the present study investigates the impact of corticosterone, a major glucocorticoid stress hormone, on the early stages of embryogenesis when somite segmentation and patterning start to appear concomitantly.

Unlike previous studies, which focus on maternal stress in mammalian systems, our work employs a controlled in ovo chick embryo model allowing direct corticosterone exposure and precise dosage regulation. This experimental design enables isolation of embryonic effects from maternal physiological influences. Besides respecting the 3Rs in animal experimentation [16], the chick embryo is also considered an established vertebrate research model due to its notable similarity to the human embryo at the molecular, cellular and anatomical levels [17]. Additionally, the chick embryo has been commonly used in developmental biology [18,19] and in reproductive toxicology research [20,21,22,23].

We provide preliminary findings that elevated corticosterone potentially affects development during the timeline of somitogenesis by downregulating key components of the SHH pathway. Moreover, alterations in hypoxia-related gene expression may provide a basis for further investigation of possible interactions between stress-related responses, hypoxia-associated pathways and SHH signalling during early development.

## 2. Materials and Methods

### 2.1. Embryo Handling and Treatment Procedure

#### 2.1.1. Reception and Cleaning of Eggs

Freshly fertilized specific pathogen-free (SPF) White Leghorn chick (*Gallus gallus domesticus*) eggs were received from Lohmann Tierzucht GmbH, Cuxhaven, Germany and cleaned with a tissue dampened with distilled water to remove surface debris. Once cleaned, the eggs were placed in a humidified incubator at 37.5 °C and 70–80% relative humidity and positioned vertically with the air sac facing upward. The first day of incubation was designated as developmental day 0.

#### 2.1.2. Windowing of Eggs

After completing 48 h of incubation (developmental day 2), the eggs were removed from the incubator and disinfected with 70% ethanol. All subsequent procedures were conducted under sterile conditions within a 15 min time frame. A small aperture was made on the side of each eggshell, and 4 mL of albumin was removed using a sterile syringe to lower the yolk, facilitating visualization of the embryo. The aperture was sealed with Tegaderm™ 3M micropore medical tape (St. Paul, MN, USA). Afterwards, a window was created at the top above the air sac.

#### 2.1.3. Treatment of Eggs

Following windowing, the eggs were randomly assigned to either a control group or a corticosterone-treated group. Control embryos (*n* = 55) received 0.5% DMSO, while treated embryos (*n* = 52) were injected in vivo with 15 µg corticosterone (dissolved in 120 µL of the respective solution) directly into the yolk sac (Supplementary Material, Video S1) The final 0.5% DMSO concentration was selected because it is widely used as a solvent control in chick embryo studies and is generally considered non-toxic at this concentration. After treatment, the window was sealed with Tegaderm™ 3M micropore medical tape. The eggs were returned to the incubator for an additional 4 h.

#### 2.1.4. Dissection and Observation of Embryos

After an additional 4 h of incubation (total incubation time = 52 h; developmental day 2), eggs were cracked open into Petri dishes. The 4 h exposure period was chosen to investigate immediate early responses to acute corticosterone exposure while minimizing secondary developmental effects that may arise after prolonged incubation.

At this point, 39 embryos from the control group and 43 embryos from the corticosterone-treated group were viable. For each embryo, excess thick albumin was removed using tissue paper and a small square of yolk sac containing the embryo was excised with scissors [24]. The embryo was carefully lifted from the yolk using a spatula spoon. The embryo was then transferred to Petri dishes containing phosphate-buffered saline (PBS) to remove residual yolk and was mounted on microscope slides for macroscopic examination under an inverted microscope.

Inter-somitic distances were measured between the first five visible pairs of somites immediately posterior to the amnion fold using ImageJ 1.54g software. Measurements were performed on standardized microscopic images obtained under identical magnification settings.

After macroscopic observation, 22/39 control and 28/43 corticosterone-treated embryos were placed in Eppendorf tubes and frozen at −80 °C for subsequent RT-qPCR analysis. Of the viable embryos, 17 control embryos and 15 corticosterone-treated embryos were fixed in 4% PFA and processed for histopathological evaluation.

**Figure 1:**
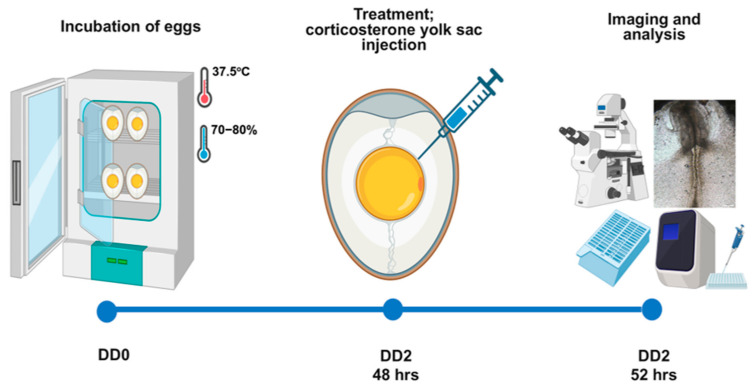
Graphical summary of the methodology (embryo upon corticosterone treatment). BioRender.com. Created in Biorender. Schneider-Stock, R. (2026) https://BioRender.com/ys6spmp (accessed on 20 May 2026).

A timeline of the methodology is represented in Figure 1.

### 2.2. Histopathological Examination

For the histopathological examination, overnight 4% PFA-fixed embryos were processed routinely, serially sectioned longitudinally at 5 μm thick and stained with haematoxylin and eosin (H&E) according to standard protocols and scanned by slide scanner P1000 (3DHistech, Sysmex Hamburg). Images were analyzed via slide viewer 2.4 (3DHistech) software, 40× magnification.

#### 2.2.1. IHC

Longitudinal sections of PFA-fixed embryos were processed routinely and incubated with anti-phospho-Histone H3 (pHH3; Biocare Medical, Pacheco, CA, USA; #CP404C, 1:100) and γH2AX (Abcam, Cambridge, UK; #ab26350, 1:2000) according to the manufacturer’s instructions and scanned by slide scanner P1000 (3DHistech). Positive cells were quantified by counting brown labelled nuclei per 0.025 mm^2^ neural tube and per full cross-section of the first five somite pairs. Images were analyzed via slide viewer 2.4 (3DHistech) software, 40× magnification.

#### 2.2.2. TUNEL-Assay

Apoptotic cells were detected in sections using the In Situ Death Detection Kit (Sigma Aldrich, St. Louis, MO, USA, #11684795910) according to the manufacturer’s instructions. Sections were counterstained with DAPI (Sigma Aldrich, #MBD0015, 1:1000), imaged by fluorescence microscope Nikon Eclipse Ti-S (Tokyo, Japan) with an exposure time of 3 s for GFP and auto setting for DAPI. Qualitative analysis was performed via ImageJ software, 20× magnification.

Appropriate positive control tissues were included for γH2AX and TUNEL staining and are shown in Supplementary Figure S1.

#### 2.2.3. Quantitative Real-Time PCR (qPCR)

RT-qPCR analysis was conducted on the frozen embryos for the gene expression of *SHH*, *GLI1*, *PTCH1*, *TGFβ4* and *HIF2α,* normalized to the reference gene, *GAPDH*. Total RNA was extracted and purified from embryos using the RNeasy Mini Kit (Qiagen in Venlo, NL; #74106) according to the manufacturer’s instructions. Briefly, primers for RT-qPCR were designed specifically for each target gene (Supplementary Table S1). Glyceraldehyde-3-phosphate dehydrogenase (GAPDH) was used as the reference gene owing to its stable expression in chicken embryonic tissues. The delta C_q_ values (ΔC_q_) for all the target samples were normalized to the reference gene of their corresponding sample. Relative transcript abundance was calculated using the 2^−ΔC_q_^ and ΔC_q_ expression = 2^−ΔC_q_^.

### 2.3. Statistical Analysis

All statistical analyses were performed using GraphPad Prism 10. Prior to statistical analysis, data distribution was assessed using the Shapiro–Wilk normality test. As several datasets did not meet the assumptions of normal distribution, comparisons between groups were performed using the non-parametric Mann–Whitney U test. Data are presented as median ± 95% confidence interval (CI). Statistical significance is indicated as follows: **** *p* < 0.0001; ** *p* < 0.01; * *p* < 0.05; ns, not significant.

## 3. Results

### 3.1. Assessment of Chick Embryo Viability

**Figure 2:**
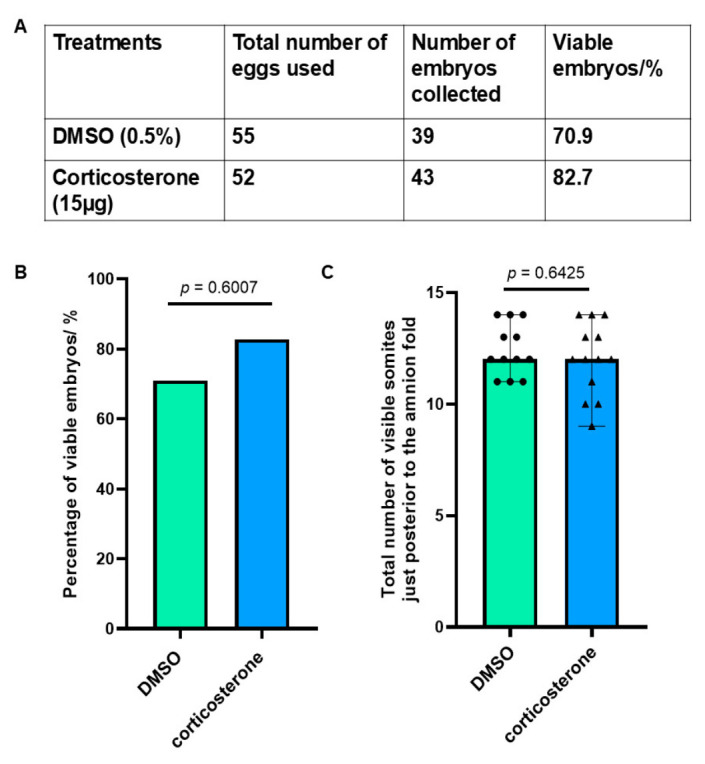
Evaluation of viability and developmental stages. (**A**,**B**) Percentage of viable embryos at the end of the experiment; (**C**) total number of somites posterior to the amnion fold to provide the developmental stage of embryos; statistical analysis has been done using Graph Pad Prism 10. Chi-Squared test was used to test for percentage viability. The Mann–Whitney U test was used to test for differences in the number of somites between control and corticosterone-treated groups.

At the end of the incubation period (after completing 52 h of incubation), viability was evaluated in both the control and corticosterone-treated groups (Figure 2A). Out of the 55 embryos from the control group, 39 remained viable. Similarly, in the corticosterone-treated group, 43 out of the 52 were found to be viable. Non-viable embryos were not subjected to further histological or molecular analyses.

Statistical analysis using the Chi-Squared test revealed no statistically significant difference in viability of the embryos between the control and corticosterone-treated groups (χ^2^ value = 0.274 and *p* = 0.6007) (Figure 2B).

### 3.2. Embryonic Stage and Macroscopic Evaluation

Due to partial obscuration of the anterior somites by the growth of cranial regions, it was only possible to accurately observe an average of 12 segmented pairs of somites just posterior to the amnion fold. The arc shaped amnion fold was used as a reliable anatomical reference point and ensured consistent evaluation across all embryos. The number of somites present in each embryo from this referral point was then counted. Consequently, the assignment of the embryonic stage was based on the number of visibly segmented somites, together with distinct morphological features observed in the cranial regions such as the precise appearance of the forebrain, midbrain and hindbrain, rather than absolute somite count. Using these combined criteria, the developmental stage of embryos was evaluated to correspond to stage HH12–13, which corresponds to a critical window of active somitogenesis [25] (Figure 2C).

**Figure 3:**
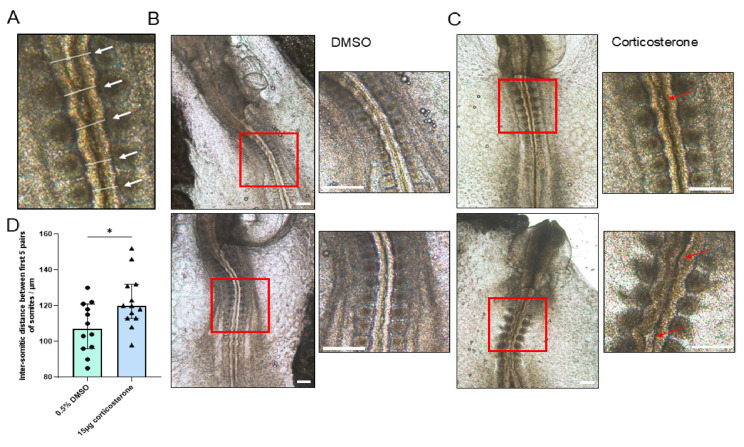
Macroscopic evaluation of embryo development. (**A**) Representative image illustrating segmentation used for somite distance measurements. (**B**) Control embryos (0.5% DMSO) showing normal flexion; the right panel displays an enlarged view of the red boxed area. (**C**) Corticosterone-treated embryos (15 µg) exhibited potential impairment in somite phenotype (red arrows). (**D**) Quantification of the inter-somitic distance between the first five pairs of somites (* *p* < 0.05, Mann–Whitney U test). Data are presented as median ± 95% CI. Scale bars: 200 µm (**B**,**C**). [Control, *n* = 12; corticosterone, *n* = 13].

Embryos were examined under an inverted microscope, and representative images were taken (Figure 3A). Additionally, we measured the inter-somitic distances between the first five pairs of somites located just posterior to the amnion fold, in both control and corticosterone-treated embryos (15 µg). The difference in inter-somitic distances between the control and corticosterone-treated embryos was statistically significant (*p* = 0.0309) (Figure 3B–D), whereby the embryos exposed to 15 µg corticosterone showed increased inter-somitic distances, indicating that increased corticosterone exposure may have a visible impact on the segmentation process of the somites at this developmental stage. Distances were measured as absolute values between adjacent somites and were not normalized to embryo length or somite size. Since embryos in both groups were assigned to the same developmental stage (HH12-13), comparisons were performed within a morphologically comparable developmental window.

### 3.3. Microscopic-Histopathological Evaluation

**Figure 4:**
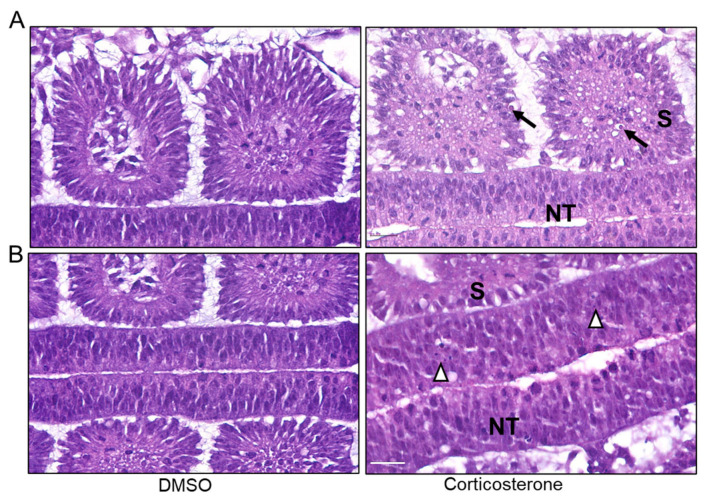
Representative photomicrographs of the neural tube (NT) and adjacent somites (S) of DMSO- or corticosterone-treated embryos: (**A**) there are occasional single, round to oval, markedly small, shrunken, bright eosinophilic circular structures (apoptotic bodies) (arrows) in somites, and (**B**) occasional karyorrhectic debris (arrowhead) observed in the neural tube. Hematoxylin and eosin (H&E) stainings were scanned by a slide scanner P1000 (3DHistech) and analyzed via slide viewer 2.4 (3DHistech) software, 40× magnification. Scale bar 20 µm.

The examination of haematoxylin and eosin (H&E) staining sections indicated isolated morphological features suggestive of cellular degeneration or cell death rather than widespread apoptotic cell death in corticosterone-treated embryos (Figure 4A,B), which was not observed in the control group.

**Figure 5:**
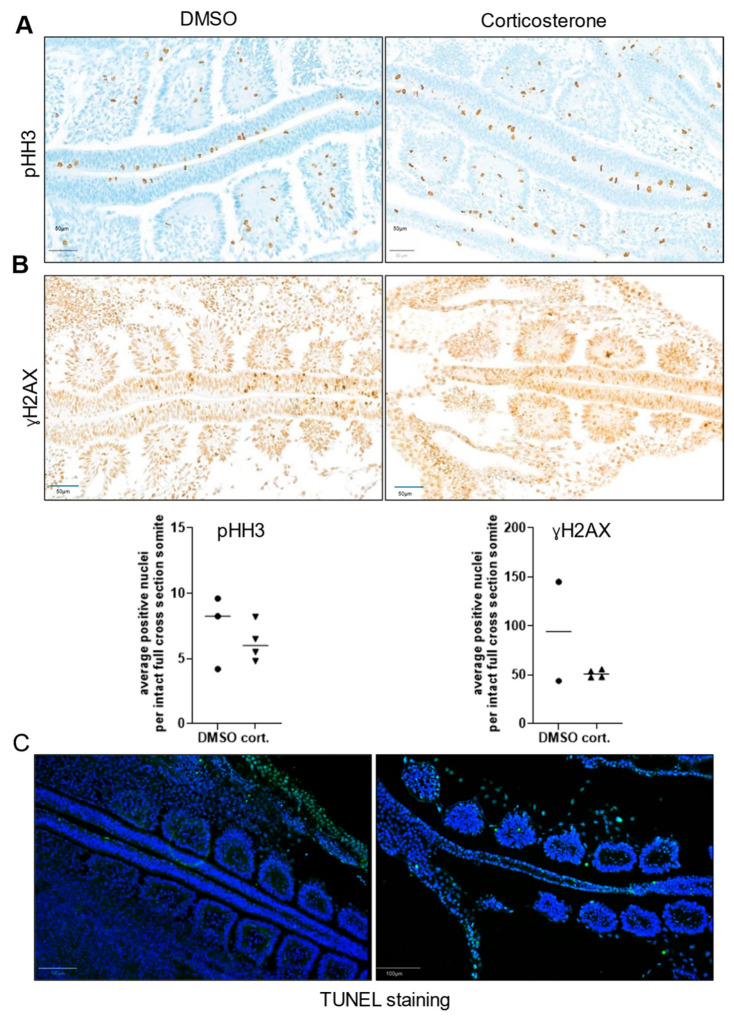
Representative photomicrographs of the neural tube and associated somites of DMSO- or 15 µg corticosterone-treated embryos (**A**,**B**). No significant differences were found in pHH3 and ɣH2AX expression in cells of the neural tube and somites between controls and treated embryos. Positive nuclei, labelled by brown granules were counted per somite and per 0.025 mm^2^ neural tube. Quantified data are presented using GraphPad Prism 10. Sections were scanned by a slide scanner P1000 (3DHistech) and analyzed via slide viewer 2.4 (3DHistech) software, 40× magnification. Scale bar 50 µm. (**C**) Single apoptotic cells were identified in controls and treated embryos. TUNEL staining was imaged by Nikon Eclipse Ti-S microscope with an exposure time of 3 s for GFP and auto setting for DAPI, which were analyzed via ImageJ software, 20× magnification, Scale bar 100 µm. Green signals denote TUNEL+ cells, and blue staining (DAPI) represents nuclei.

Since antibodies suitable for avian tissue are extremely limited, we selected three markers that target DNA. TUNEL staining revealed occasional positive cells in both control and corticosterone-treated embryos. Owing to variability in section orientation and anatomical level, TUNEL staining was evaluated qualitatively. No obvious increase in TUNEL-positive cells was observed in corticosterone-treated embryos compared with controls. Actively dividing cells labelled pHH3 were seen on the apical neuroepithelial surface of the neural tube of control and treated embryos, indicating preserved proliferative architecture. Further, the number of actively dividing cells and cells with DNA double-strand breaks labelled by γH2AX did not differ significantly between control and corticosterone-treated groups (Figure 5A–C). Positive controls for γH2AX and TUNEL staining are given in Supplementary Figure S1.

Of note, embryos that did not survive until the experimental endpoint were not available for systematic histological or molecular evaluation. Consequently, the causes of embryonic death could not be determined. Although no statistically significant differences in viability were observed between groups, it cannot be excluded that embryos exhibiting the most severe corticosterone-induced developmental alterations failed to survive until analysis.

### 3.4. Molecular Evaluation

Furthermore, we analyzed the relative mRNA expression of key genes from the Sonic hedgehog (SHH) pathway—*PTCH1*, *SHH*, and *GLI1*—as well as *HIF2α* and *TGFβ4*. Expression levels were normalized to *GAPDH*. The gene expression levels were determined as the mean C_q_ value from three technical replicates, and each data point represents the mean C_q_ value of one embryo.

**Figure 6:**
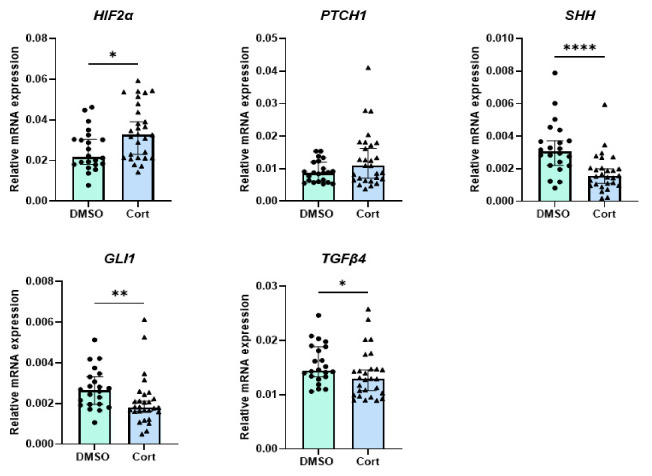
Relative mRNA expression of specific genes *HIF2α*, *PTCH1*, *SHH*, *GLI1*, and *TGFβ4* in the developing chicken embryos at 52 h. **** *p* < 0.0001, ** *p* < 0.01, * *p* < 0.05, Mann–Whitney U test (*HIF2α*: *p* = 0.01, *PTCH1*: *p* = 0.164, *SHH*: *p* < 0.0001, *GLI1*: *p* = 0.0057, *TGFβ4*: *p* = 0.0389). Data are presented as Median ± 95% CI. Each data point represents one embryo (PCR was performed in 3 technical replicates); cort = corticosterone. Relative mRNA expression levels are shown, normalized to *GAPDH*. Each data point represents one embryo. [Control, *n* = 22; corticosterone, *n* = 28].

A significant decrease in the relative mRNA expression of *SHH* and *GLI1* was observed in corticosterone-treated embryos (Figure 6). In contrast, *HIF2α* expression was significantly increased in corticosterone-treated embryos, whereas *TGFβ4* expression showed a significant reduction (Figure 6). Embryos treated with corticosterone showed a trend toward increased *PTCH1* expression compared with controls; however, this difference did not reach statistical significance (Figure 6).

## 4. Discussion

The SHH signalling pathway is required to maintain the timely operation of the molecular clock and the correct pace of somite formation [8]. Here, we demonstrate that exposure to an acute high concentration of corticosterone (15 µg) during early chick embryogenesis correlates with disruptions in somite phenotypes and potential patterning. Although the 15 µg corticosterone dose has been previously described as a pathophysiological exposure level in day 6 chick embryos [26], its biological equivalence at earlier developmental stages remains uncertain. This dose was selected because it represents an established in ovo corticosterone exposure model and enabled the investigation of acute stress hormone effects without causing embryonic lethality in our experimental setting. Importantly, embryonic sensitivity to corticosterone is likely to vary with developmental stages. For instance, ^3^H-corticosterone-binding capacity to cytosolic proteins differs substantially between 2-, 3- and 4-day-old chick embryos, suggesting stage-dependent responsiveness to glucocorticoid exposure [27]. Therefore, the present study should be interpreted as investigating the effects of acute high-dose corticosterone challenge during early somitogenesis rather than modelling a precisely defined maternal stress equivalent.

Furthermore, following yolk sac administration, the pharmacokinetics of corticosterone uptake and distribution during this early developmental stage are not fully characterized. Consequently, the proportion of the administered dose that reached embryonic tissues after the 4 h exposure period remains unknown. This limitation should be considered when interpreting the magnitude of the observed molecular and morphological effects.

In this study, sections were made along the dorsal line of the developing embryo, which allowed the observations of marked increases in the distance between the first five pairs of somites just posterior to the amnion fold, in the corticosterone-treated embryos. These results provide a first indication of potential impairment in normal somite phenotype. In the present morphometric analysis, all inter-somitic distance measurements were performed in a standardized manner between the first five somite pairs immediately posterior to the amnion fold, thereby ensuring that equivalent anatomical regions were compared between embryos. Nevertheless, inter-somitic distances were evaluated as absolute measurements and were not normalized to embryo length or somite dimensions. Although embryos from both groups exhibited comparable Hamburger–Hamilton developmental stages and similar somite numbers, subtle differences in overall embryonic growth, tissue orientation, or processing cannot be completely excluded. Therefore, the observed increase in inter-somitic distances should be interpreted with appropriate caution. Future studies incorporating normalized morphometric parameters and complementary imaging approaches may further refine the assessment of corticosterone-associated alterations in somite organization.

Histopathological examination revealed occasional cells displaying morphological features consistent with apoptosis, including hypereosinophilic shrunken cell bodies and karyorrhectic nuclear debris. In contrast, these observations were not accompanied by a significant increase in TUNEL-positive cells or γH2AX staining. These findings are not necessarily contradictory, as histo-morphological evaluation and TUNEL staining detect different biological aspects and stages of cell death. Moreover, alternative forms of cell death that are not efficiently detected by TUNEL staining cannot be excluded. Because embryonic sections varied with respect to anatomical level and orientation, quantitative assessment of TUNEL-positive cells was not performed. Therefore, the present data should be interpreted as indicating the absence of widespread apoptotic cell death while not excluding subtle or localized corticosterone-associated cellular degeneration.

Previous studies have suggested that glucocorticoids can modulate Hedgehog signalling during development [28,29]. Recent findings indicate that cortisol may directly interfere with Smoothened activation by competing with cholesterol binding in mouse lung fibroblasts [30], providing a potential molecular mechanism for stress hormone-induced disruption of SHH signalling. While in ovo, corticosterone models have been employed to simulate systemic stress exposure and have been shown to impair early skin development [26], the specific effects on the SHH pathway during early embryogenesis have not been thoroughly documented.

In the absence of SHH ligand, PTCH1 represses Smoothened, thereby inhibiting downstream signalling and preventing activation of GLI-dependent transcription [31]. When the SHH ligand binds to PTCH1, the inhibitory effect is removed. In our study, *SHH* and *GLI1* expression were significantly reduced following corticosterone exposure, but *PTCH1* expression did not differ significantly between groups. Since *PTCH1* is a transcriptional target of Sonic hedgehog signalling [32], the absence of a corresponding decrease may reflect the complexity of *PTCH1* regulation during early embryogenesis rather than activation of the pathway.

Importantly, *PTCH1* exhibits highly dynamic and spatially restricted expression patterns within the developing neural tube and paraxial mesoderm. Since mRNA was extracted from whole embryos, localized changes in *PTCH1* expression may have been masked by surrounding tissues not directly affected by corticosterone exposure. Therefore, the present bulk RT-qPCR approach may not fully capture region-specific alterations in SHH signalling pathway activity.

The concurrent downregulation of *SHH*, *GLI1* and *TGFβ4*, along with increases in inter-somitic distances, further suggests a corticosterone-induced disruption of somite phenotype. Furthermore, based on our findings of the significant upregulation of the expression levels of *HIF2α*, we hypothesize that in vivo exposure to high corticosterone levels may impact hypoxic conditions. The significant increase in *HIF2α* expression suggests that corticosterone exposure may influence embryonic oxygen homeostasis during this critical developmental window. Consequently, the observed downregulation of *SHH* and *GLI1* may arise through non-mutually exclusive mechanisms. First, corticosterone may directly modulate the SHH signalling pathway through glucocorticoid receptor-dependent pathways, as previous studies have demonstrated interactions between glucocorticoid and Hedgehog signalling networks [33]. Since glucocorticoid receptor binding can remodel chromatin accessibility and alter transcriptional activity [15], elevated corticosterone levels may directly influence the expression of developmental signalling genes, including components of the SHH pathway. Interestingly, direct crosstalk between glucocorticoid signalling and the Hedgehog pathway has been described previously, as selected glucocorticoids were found to activate Smoothened and downstream *GLI*-dependent transcription [34]. Second, corticosterone may indirectly affect SHH activity through hypoxia-associated responses. HIF signalling is known to interact with developmental patterning pathways and may alter cellular differentiation programmes during embryogenesis [10,11,12].

However, because the present data do not allow discrimination between direct glucocorticoid-mediated effects and secondary hypoxia-associated mechanisms, the concurrent increase in *HIF2α* expression and decrease in *SHH* and *GLI1* expression should be interpreted as supporting a hypothesis of a possible stress–hypoxia–SHH axis during early somitogenesis. Future studies examining tissue-specific expression patterns, vascular development, and glucocorticoid receptor signalling will be required to test this hypothesis and clarify the causal relationships among these pathways. Additionally, observation periods beyond the 4 h exposure window may be warranted to confirm the developmental impacts over the long term.

In mammals, placental 11β-hydroxysteroid dehydrogenase tightly regulates embryonic and fetal exposure to maternal glucocorticoids, protecting against excess hormonal exposure and shaping developmental outcomes [35]. Disruption of this regulation, as seen in guinea pig models subjected to elevated maternal glucocorticoids, results in persistent alterations to offspring HPA axis activity and impaired fetal development [36]. Unlike mammalian models, chick embryos lack placental mediation, but the yolk sac of the chick embryo can be considered as serving as a placental barrier because it allows the transfer of stored nutrients [37] and hormones from the yolk to the developing embryo. By making use of the chick embryo model in our study and through direct yolk injection of corticosterone, precise control over both the dosage and timing of hormone exposure was achieved. This approach provides a robust platform for investigating the effects of stress hormones on early vertebrate development and offering insights that can extend to and complement mammalian studies.

## 5. Conclusions

This exploratory study shows that acute corticosterone exposure during early avian embryogenesis is associated with altered somite organization and changes in SHH pathway-related gene expression. These findings provide a basis for future mechanistic studies investigating the interplay between glucocorticoid signalling, embryonic stress responses, and developmental signalling pathways during early embryogenesis.

## Data Availability

The original contributions presented in this study are included in the article/Supplementary Materials. Further inquiries can be directed to the corresponding authors.

## Supplementary Materials

The following supporting information can be downloaded at: https://www.mdpi.com/article/10.3390/biom16071014/s1. Figure S1: Representative photomicrographs of positive controls tissues used for (A) TUNEL staining for apoptotic cells. The chicken embryo tissue (yolk sac) used as a positive control was treated with DNase (Qiagen, Germany, #79256; 3 U/ml in 100 µl) for 15 minutes at 37 °C. TUNEL staining was imaged by Nikon eclipse Ti-S microscope with an exposure time of 3 s for GFP and auto setting for DAPI, which are analyzed via ImageJ software, 20× magnification, Scale bar 100 µm. Green signals denote TUNEL+ cells and blue staining (DAPI) represent nuclei. (B) ɣH2AX labeling of double stranded DNA breaks. Multifocal brown nuclear labelling of few crypt epithelial cells in the human small intestine section indicates specificity. Sections were scanned by Slides scanner P1000 (3DHistech) and analyzed via slide viewer 2.4 (3DHistech) software, 40× magnification. Scale bar 50 µm. Table S1: Primer sequences and Melting temperatures for gene expression analysis. Video S1: The video demonstrates the process of administering 15 µg corticosterone into the yolk sac of the developing chicken embryo through injection. This method of chemical delivery ensures efficient systemic delivery of the corticosterone, allowing it to be distributed throughout the developing embryo.

## References

[1] Coussons-Read M.E. (2013). Effects of prenatal stress on pregnancy and human development: Mechanisms and pathways. Obstet. Med..

[2] Tadanki D., Kaza P.S., Meisinger E., Syed A., Johnson A., Bainbridge G., Cho M., Anigbogu C., Gupta G. (2025). Comprehensive Review of the Impact of Maternal Stress on Fetal Development. Pediatr. Discov..

[3] Guardino C.M., Schetter D., Saxbe D.E., Adam E.K., Ramey S.L., Shalowitz M.U. (2016). Diurnal salivary cortisol patterns prior to pregnancy predict infant birth weight. Health Psychol..

[4] Wadhwa P.D., Entringer S., Buss C., Lu M.C. (2011). The Contribution of Maternal Stress to Preterm Birth: Issues and Considerations. Clin. Perinatol..

[5] Chau K.F., Springel M.W., Broadbelt K.G., Park H.Y., Topal S., Lun M.P., Mullan H., Maynard T., Steen H., LaMantia A.S. (2015). Progressive Differentiation and Instructive Capacities of Amniotic Fluid and Cerebrospinal Fluid Proteomes following Neural Tube Closure. Dev. Cell..

[6] Miao Y., Pourquié O. (2024). Cellular and molecular control of vertebrate somitogenesis. Nat. Rev. Mol. Cell Biol..

[7] Boareto M., Tomka T., Iber D. (2021). Positional information encoded in the dynamic differences between neighboring oscillators during vertebrate segmentation. Cells Dev..

[8] Resende T.P., Ferreira M., Teillet M.A., Tavares A.T., Andrade R.P., Palmeirim I. (2010). Sonic hedgehog in temporal control of somite formation. Proc. Natl. Acad. Sci. USA.

[9] Bijlsma M.F., Groot A.P., Oduro J.P., Franken R.J., Schoenmakers S.H., Peppelenbosch M.P., Spek C.A. (2009). Hypoxia induces a hedgehog response mediated by HIF-1alpha. J. Cell Mol. Med..

[10] Hwang J.M., Weng Y.J., Lin J.A., Bau D.T., Ko F.Y., Tsai F.J., Tsai C.H., Wu C.H., Lin P.C., Huang C.Y. (2008). Hypoxia-induced compensatory effect as related to Shh and HIF-1alpha in ischemia embryo rat heart. Mol. Cell Biochem..

[11] Sims J.R., Lee S.W., Topalkara K., Qiu J., Xu J., Zhou Z., Moskowitz M.A. (2009). Sonic hedgehog regulates ischemia/hypoxia-induced neural progenitor proliferation. Stroke.

[12] Yan Y., Liu F., Han L., Zhao L., Chen J., Olopade O.I., He M., Wei M. (2018). HIF-2α promotes conversion to a stem cell phenotype and induces chemoresistance in breast cancer cells by activating Wnt and Notch pathways. J. Exp. Clin. Cancer Res..

[13] Echevarría-Andino M.L., Allen B.L. (2020). The hedgehog co-receptor BOC differentially regulates SHH signaling during craniofacial development. Development.

[14] Xu S., Shi J., Shen Y., Chen X., Pourbozorg G., Wang G., Yang X., Cheng X. (2024). Prenatal Maternal Stress Suppresses Embryonic Neurogenesis via Elevated Glucocorticoid Levels. Endocrinology.

[15] Bothe M., Buschow R., Meijsing S.H. (2021). Glucocorticoid signaling induces transcriptional memory and universally reversible chromatin changes. Life Sci. Alliance.

[16] Fischer D., Fluegen G., Garcia P., Ghaffari-Tabrizi-Wizsy N., Gribaldo L., Huang R.Y.J., Rasche V., Ribatti D., Rousset X., Pinto M.T. (2022). The CAM Model Q&A with experts. Cancers.

[17] Wallis J.W., Aerts J., Groenen M.A., Crooijmans R.P.M.A., Layman D., Graves T.A., Scheer D.E., Kremitzki C., Fedele M.J., Mudd N.K. (2004). A physical map of the chicken genome. Nature.

[18] Le Douarin N.M., Dieterlen-Lièvre F. (2013). How studies on the avian embryo have opened new avenues in the understanding of development: A view about the neural and hematopoietic systems. Dev. Growth Differ..

[19] Vergara M.N., Canto-Soler M.V. (2012). Rediscovering the chick embryo as a model to study retinal development. Neural Dev..

[20] Bjørnstad S., Austdal L.P.E., Roald B., Glover J.C., Paulsen R.E. (2015). Cracking the Egg: Potential of the Developing Chicken as a Model System for Nonclinical Safety Studies of Pharmaceuticals. J. Pharmacol. Exp. Ther..

[21] Goodfellow F.T., Tesla B., Simchick G., Zhao Q., Hodge T., Brindley M.A., Stice S.L. (2016). Zika Virus Induced Mortality and Microcephaly in Chicken Embryos. Stem Cells Dev..

[22] Wachholz G.E., Rengeal B.D., Vargesson N., Fraga L.R. (2021). From the Farm to the Lab: How Chicken Embryos Contribute to the Field of Teratology. Front. Genet..

[23] Zosen D., Hadera M.G., Lumor J.S., Andersen J.M., Paulsen R.E. (2021). Chicken embryo as animal model to study drug distribution to the developing brain. J. Pharmacol. Toxicol. Methods.

[24] Psychoyos D., Finnell R. (2008). Method for culture of early chick embryos ex vivo (New culture). J. Vis. Exp..

[25] Hamburger V., Hamilton H.L. (1951). A series of normal stages in the development of the chick embryo. J. Morphol..

[26] Gellisch M., Bablok M., Divvela S.S.K., Moroscan- Puopolo G., Brand-Saberi B. (2023). Systemic Prenatal Stress Exposure through Corticosterone Application Adversely Affects Avian Embryonic Skin Development. Biology.

[27] Jelínek R., Pavlík A., Peterka M. (1983). Glucocorticoid receptor-mediated teratogenesis in the chick embryo. Teratog. Carcinog. Mutagen..

[28] Finco I., LaPensee C.R., Krill K.T., Hammer G.D. (2015). Hedgehog signaling and steroidogenesis. Annu. Rev. Physiol..

[29] Heine V.M., Rowitch D.H. (2009). Hedgehog signaling has a protective effect in glucocorticoid-induced mouse neonatal brain injury through an 11βHSD2-dependent mechanism. J. Clin. Investig..

[30] Lu L., Liu Y.D., Liu D.Y., Jin R., Qu Z.H. (2025). Analysis of Fibroblast Growth Factor 2 Impact and Mechanism on Broncho-Pulmonary Dysplasia. Int. Arch. Allergy Immunol..

[31] Krishnan M., Kumar S., Kangale L.J., Ghigo E., Abnave P. (2021). The Act of Controlling Adult Stem Cell Dynamics: Insights from Animal Models. Biomolecules.

[32] Rahnama F., Shimokawa T., Lauth M., Finta C., Kogerman P., Teglund S., Toftgard R., Zaphiropoulos P.G. (2006). Inhibition of GLI1 gene activation by Patched1. Biochem. J..

[33] Bongiovanni D., Tosello V., Saccomani V., Dalla Santa S., Amadori A., Zanovello P., Piovan E. (2020). Crosstalk between Hedgehog pathway and the glucocorticoid receptor pathway as a basis for combination therapy in T-cell acute lymphoblastic leukemia. Oncogene.

[34] Wang J., Lu J., Bond M.C., Chen M., Ren X., Lyerly H.K., Barak L.S., Chen W. (2010). Identification of select glucocorticoids as Smoothened agonists: Potential utility for regenerative medicine. Proc. Natl. Acad. Sci. USA.

[35] Duthie L., Reynolds R.M. (2013). Changes in the Maternal Hypothalamic-Pituitary-Adrenal Axis in Pregnancy and Postpartum: Influences on Maternal and Fetal Outcomes. Neuroendocrinology.

[36] Kapoor A., Matthews S.G. (2008). Prenatal stress modifies behavior and hypothalamic-pituitary-adrenal function in female guinea pig offspring: Effects of timing of prenatal stress and stage of reproductive cycle. Endocrinology.

[37] Shibata M., Makihara N., Iwasawa A. (2023). The Yolk Sac’s Essential Role in Embryonic Development. Rev. Agric. Sci..

