# Supplementary material for: High Corticosterone Affects Somite Development During Early Avian Embryogenesis
Source: Biomolecules. 2026 Jul 11;16(7):1014. doi: 10.3390/biom16071014 (PMC13406671; doi:10.3390/biom16071014)
Supplement: Supplementary file 1 [file biomolecules-16-01014-s001.zip › supplementary material.pdf]

**Supplementary material**

Supplementary Movie S1

The video demonstrates the process of administering 15µg corticosterone into the yolk sac of the developing chicken embryo through injection. This method of chemical delivery ensures efficient systemic delivery of the corticosterone, allowing it to be distributed throughout the developing embryo.

Supplementary Figure S1

Representative photomicrographs of positive controls tissues used for (A) TUNEL staining for apoptotic cells. The chicken embryo tissue (yolk sac) used as a positive control was treated with DNase (Qiagen, Germany, #79256; 3 U/ml in 100 µl) for 15 minutes at 37°C. TUNEL staining was imaged by Nikon eclipse Ti-S microscope with an exposure time of 3 s for GFP and auto setting for DAPI, which are analyzed via ImageJ software, 20× magnification, Scale bar 100 µm. Green signals denote TUNEL+ cells and blue staining (DAPI) represent nuclei.

(B) ɣH2AX labeling of double stranded DNA breaks. Multifocal brown nuclear labelling of few crypt epithelial cells in the human small intestine section indicates specificity. Sections were scanned by Slides scanner P1000 (3DHistech) and analyzed via slide viewer 2.4 (3DHistech) software, 40× magnification. Scale bar 50 µm.
