# Supplementary material for: High Corticosterone Affects Somite Development During Early Avian Embryogenesis
Source: Biomolecules. 2026 Jul 11;16(7):1014. doi: 10.3390/biom16071014 (PMC13406671; doi:10.3390/biom16071014)
Supplement: Supplementary file 1 [file biomolecules-16-01014-s001.zip › Supplementary table S1.pdf]

**Supplementary Table S1**

**Primer sequences and Melting temperatures for gene expression analysis**

| Markers | Primers sequences (5′-3′); F: Forward, R: Reverse | Melting temperature (°C) |
|---|---|---|
| *GAPDH* | F: CCT CTC TGG CAA AGT CCA AG<br>R : GGT CAC GCT CCT GGA AGA TA | 80 |
| *SHH* | F: TGC TAG GGA TCG GTG GAT AG<br>R: ACA AGT CAG CCC AGA GGA GA | 80 |
| *GLI-1* | F: CCT CTG ACA GCC AAA TAC CCA G<br>R: TTT CCC ATC CTC CTT CTC CAG | 82.5 |
| *PTCH-1* | F: TGC CAG CCT ATC ACT ACT GTG<br>R: CAT TCG ACA TCC TGA AGC TC | 81.5 |
| *HIF-2α* | F: ATC AAG TTC CCC CTC AGG AC<br>R: TGT TGC AAT GCT TGC TCT TC | 79.5 |
| *TGF-β4* | F: CAC CGA CTA CTG CTT CGG C<br>R: GTC GGC GCT CCA GAT GTA C | 84.5 |
