# Supplementary figures and images for: High Corticosterone Affects Somite Development During Early Avian Embryogenesis
Source: Biomolecules. 2026 Jul 11;16(7):1014. doi: 10.3390/biom16071014 (PMC13406671; doi:10.3390/biom16071014)

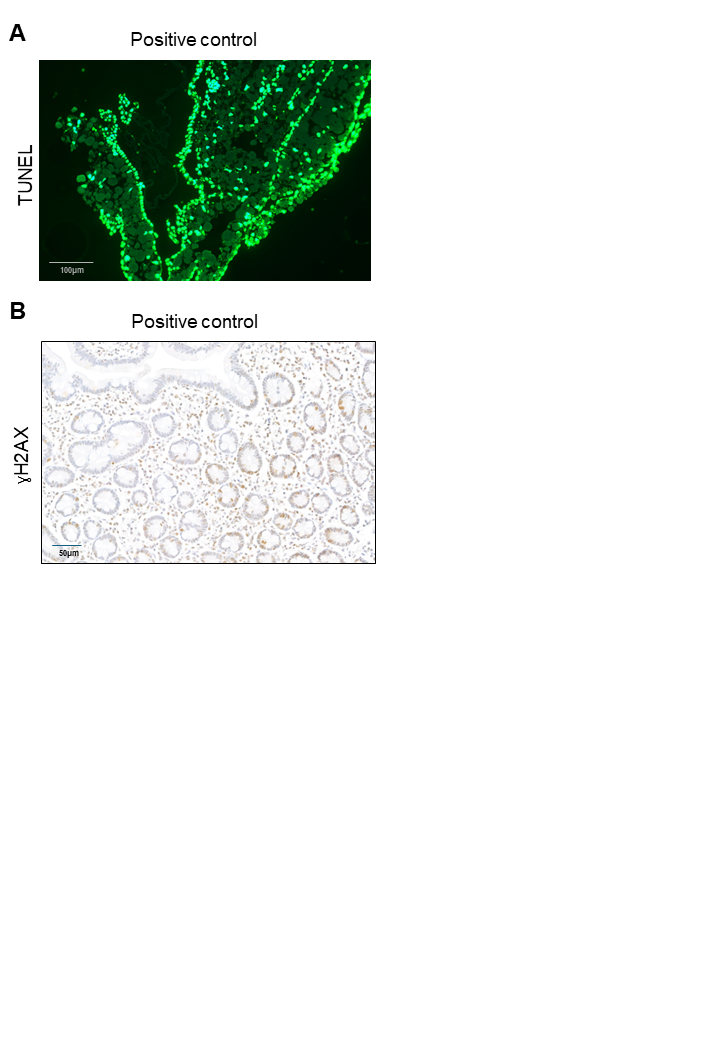

Supplement: Supplementary file 1 [file biomolecules-16-01014-s001.zip › Suppl. Figure S1.png]
